# Image Quality and Lesion Detectability with Low-Monoenergetic Imaging: A Study of Low-Concentration Iodine Contrast in Hepatic Multiphase CT for Chronic Liver Disease

**DOI:** 10.3390/tomography11060066

**Published:** 2025-06-04

**Authors:** Jae En Kim, Yewon Lim, Jin Sil Kim, Hyo Jeong Lee, Jeong Kyong Lee, Hye Ah Lee

**Affiliations:** 1 College of Medicine, Ewha Womans University, Seoul 07804, Republic of Korea; siamese49@ewhain.net; 2 Department of Radiology, Ewha Womans University Mokdong Hospital, College of Medicine, Ewha Womans University, Seoul 07985, Republic of Korea; jennyannalim@gmail.com (Y.L.); hjleerad@ewha.ac.kr (H.J.L.); kyongmd@ewha.ac.kr (J.K.L.); 3 Clinical Trial Center, Mokdong Hospital, Ewha Womans University, Seoul 07985, Republic of Korea; khyeah@ewha.ac.kr

**Keywords:** radiation dosage, radiography, dual-energy scanned projection, iodine, contrast media, tomography

## Abstract

Background: This study aimed to evaluate whether low-concentration iodine contrast-enhanced multiphase low-monoenergetic computed tomography (LCLM CT; 270 mg I/mL, 40 keV) is non-inferior to standard-dose computed tomography (SDCT; 350 mg I/mL) in image quality and lesion detectability for chronic liver disease patients. Methods: Sixty-seven patients underwent both protocols. Image quality was assessed using a 5-point scale with a non-inferiority margin of −0.5. Quantitative metrics included signal-to-noise ratio (SNR) and contrast-to-noise ratio (CNR). Lesion detectability was evaluated using jackknife free-response receiver operating characteristic (JAFROC) analysis with a −0.1 margin. Results: LCLM CT reduced iodine dose per kilogram by 21.9%. Despite higher image noise, it achieved higher CNR for the aorta and hepatic lesions, as well as superior hepatic artery clarity. Image quality was non-inferior (difference: −0.119; 95% CI: −0.192 to −0.047), and lesion detectability (FOM: 0.744 vs. 0.721; difference: 0.023; 95% CI: −0.170 to 0.218) also showed non-inferiority. Conclusions: LCLM CT maintains diagnostic performance and improves vascular contrast while reducing iodine burden, supporting its clinical utility in longitudinal HCC surveillance.

## 1. Introduction

Worldwide, hepatocellular carcinoma (HCC) accounts for approximately 80% of all primary liver cancers [1]. Despite advancements in HCC treatment, the 5-year intrahepatic recurrence rate remains high, reaching 80% [2,3]. Therefore, continuous and intense surveillance for detecting early HCC and its recurrence is crucial for managing chronic liver disease patients [4,5,6]. Multiphase hepatic computed tomography (CT) or magnetic resonance imaging (MRI) are commonly used for HCC surveillance, with CT offering several advantages compared to MRI, such as shorter scan times and lower costs [7,8]. While contrast-enhanced ultrasound (CEUS) using microbubble-based contrast agents offers alternatives for specific patient groups, multiphase CT remains an essential imaging modality for longitudinal surveillance due to its comprehensive liver evaluation capabilities, especially in patients who have undergone multiple treatments [9,10]. However, the primary drawbacks of CT include repeated exposure to radiation and administration of iodinated contrast media.

The concern regarding iodinated contrast media largely stems from the potential for renal toxicity, which is generally correlated with the total amount of iodine delivered [11]. Consequently, recent research has focused on strategies to reduce the overall contrast media dose while maintaining diagnostic image quality, primarily by decreasing the administered volume [12,13].

An equally critical yet underexplored factor is the iodine concentration of the contrast agent itself. Higher iodine concentrations lead to increases in contrast viscosity and osmolality, increasing the risk of renal toxicity patient discomfort during administration, and potential risk of extravasation [11,14]. Therefore, using contrast agents with a lower iodine concentration offers distinct clinical advantages; it not only naturally reduces the total iodine dose but also directly mitigates the risks associated with high viscosity and osmolality, making it particularly beneficial for patients undergoing frequent CT scanning for follow-up, such as those with chronic liver disease. Despite this potential, research specifically investigating the impact of reducing iodine concentration itself is scarce. Although a prior study reported no significant differences in the portal venous phase images when using contrast with lower iodine concentration for the abdominal organs [15], the diagnostic performance of reducing iodine concentration in multiphase hepatic CT for patients with chronic liver disease remains underexplored.

Reducing iodine concentration in hepatic CT is challenging as it affects arterial enhancement, which is critical for HCC evaluation [16]. According to previous studies, low-monoenergetic imaging generated from dual-energy CT can provide improved iodine contrast compared to conventional imaging using the same iodine concentration [17] and improve the contrast-to-noise ratio (CNR), with 40–50 keV commonly used for optimal vascular visualization [18,19,20]. Notably, 40 keV has been shown to provide the highest enhancement of iodine contrast, with acceptable noise levels for hepatic imaging, particularly in the arterial phase [18,21]. This is primarily because 40 keV is close to the K-edge of iodine (33.2 keV), leading to a marked increase in iodine attenuation and a substantial rise in contrast [22]. This suggests that low-keV monoenergetic imaging may enable the reduction of contrast concentration without compromising image quality, especially in the arterial phase [23,24,25].

Therefore, this study aimed to evaluate whether the image quality and detectability of hepatic multiphase CT with low-concentration iodine contrast (270 mg I/mL) using low-monoenergetic imaging (40 keV; LCLM CT) is non-inferior to those of the standard-dose CT (high-concentration iodine contrast, 350 mg I/mL) using hybrid iterative reconstruction (SDCT) focusing on the arterial phase images in patients with chronic liver disease.

## 2. Materials and Methods

This study was conducted after receiving approval from the Institutional Review Board of Ewha Womans University Mokdong Hospital (IRB file number 2024-09-012). All the experimental methods were conducted in accordance with the relevant guidelines and regulations. The study was conducted retrospectively, with data obtained after the imaging was completed. All collected data was handled with strict confidentiality and was accessible only to the researchers. All identifiable patient information was excluded. The waiving of the need for informed consent was confirmed by the Institutional Review Board (IRB) due to the retrospective nature of the study and exclusion of identifiable information.

### 2.1. Patient Population

From June 2023 to December 2023, a total of 511 patients received a hepatic multiphase CT scan with low-concentration iodine contrast (270 mg I/mL Iohexol [Iobrix inj. 270; Taejoon Pharm Co., Ltd., Seoul, Republic of Korea]). Patients without low-monoenergetic arterial phase imaging were excluded (*n* = 273). Additionally, patients who had not undergone a prior hepatic multiphase CT with 350 mg I/mL contrast media (Iobtridol [Xenetix 350, Guerbet, Sulzbach, Germany], Iohexol [Bonorex 350, Central Medical Service, Seoul, Republic of Korea; Omnipaque 350, GE Healthcare, Little Chalfont, Milton Keynes, UK]; *n* = 151) or did not have an underlying condition identified as a risk factor for HCC, based on the 2018 version of the Liver Imaging Reporting and Data System (LI-RADS) [26] (*n* = 20), were excluded from the study. Finally, 67 eligible patients were included in the analysis. The sample size required for the evaluation of the non-inferiority of the overall image quality of the SDCT was calculated, and it was found that 41 patients were required (Appendix A).

### 2.2. Image Acquisition and Reconstruction

LCLM CTs were performed with either a 192-detector row CT scanner (SOMATOM Force; Siemens Healthcare, Forchheim, Germany) (*n* = 36) or a 128-detector row CT scanner (SOMATOM Definition Flash, Siemens Healthcare, Forchheim, Germany) (*n* = 31) with automated tube current modulation (CareDose 4D, Siemens Healthcare). The arterial phase images were obtained in dual-energy (DE) mode. The CT parameters used in this study included the following: 128 × 0.6 mm collimation, 0.6 pitch, 3 mm reconstruction interval, two separate tube voltages (80 kV and tin-filtered 150 Kv [Sn150 Kv]), and 325/163 (mAs) reference tube currents for SOMATOM Force and 32 × 0.6 mm collimation, 0.6 pitch, 3 mm reconstruction interval, two separate tube voltages (100 kV and tin-filtered 140 kV [Sn140 Kv]), and 219/170 (mAs) reference tube currents for SOMATOM Definition Flash. Monoenergetic (40 keV) images were reconstructed. In our study, 40 keV was selected as it has been shown to provide the highest enhancement of iodine contrast with acceptable noise levels for hepatic imaging, particularly in the arterial phase, which is critical for detecting hepatocellular carcinoma (HCC) [18,21]. Unenhanced scans were initially obtained. For patients weighing ≤60 kg, 110 mL of contrast (**Iobrix inj. 270; Taejoon Pharm Co., Ltd., Seoul, Republic of Korea**), equivalent to 463 mg I/kg for a 70 kg individual) was administered, and 120 mL was given to those weighing >60 kg. The contrast was intravenously administered with an automatic power injector at a rate of 3 mL/s. The arterial phase images were obtained 12 s after the contrast reached a trigger threshold of 100 HU at the abdominal aorta, using the bolus tracking technique. The portal venous phase images were obtained 80–90 s after the administration of contrast medium, while the delayed phase images were obtained 180 s after the administration of contrast medium. The portal venous phase images included the area from the lower chest to the pelvic cavity, while other phases scanned the area from the lower chest to the inferior liver pole.

For SDCT, three different multi-detector CT scanners (SOMATOM Force, SOMATOM Definition Flash, and SOMATOM Sensation 64) with automated tube current modulation (CareDose4D, Siemens Healthcare) were used for image acquisition. The same bolus triggering technique as LCLM CT was employed for all phases with 350 mg I/mL contrast (Iohexol [Bonorex 350, Central Medical Service] or Iobitridol [Xenetix 350, Guerbet] 350 mg I/mL; equivalent to 600 mg I/kg based on 70 kg). We used the mixed image acquired from a dual-energy scan to compare it with images from a single-energy scan for SOMATOM Force and SOMATOM Definition Flash. The mixed ratio for the blended image was 0.6 (60% 80 kV and 40% Sn150 kV) for SOMATOM Force and 0.5 (50% 100 kV and 50% Sn140 kV) for SOMATOM Definition Flash. A hybrid iterative reconstruction technique based on ADMIRE 3 was used for the reconstruction of the mixed images. This is a partial model-based reconstruction technique and is known to have the highest image quality for abdominal CT reconstruction [27]. Single-energy (SE) scan was used for SOMATOM Sensation 64, with collimation of 32 × 0.6 mm, a pitch of 1, a reconstruction interval of 3 mm, a tube voltage of 100 kV, and a reference tube current of 210 mAs for the imaging parameters. The characteristics of the different CT scanners are presented in Supplementary Table S1.

### 2.3. Qualitative Image Analysis

A total of 67 pairs of arterial phase CT scans (LCLM CT and SDCT) were reviewed by two abdominal radiologists (*J.S.K.* with 13 years of experience in abdominal CT interpretation and *H.J.L.* with 7 years of such experience). The assessments were conducted using our clinical picture archiving and communication system (PACS, INFINITT Healthcare, Seoul, Republic of Korea). The radiologists evaluated the overall image quality, hepatic artery clarity, image contrast, and image noise on a 5-point scale, where higher scores indicated better image quality (Table 1). These evaluation criteria were based on grading systems established in previous studies [25,28].

### 2.4. Quantitative Image Analysis

One researcher (*J.E.K.*) drew 3 regions of interest (ROIs) in the liver, aorta, and subcutaneous fat of the anterior abdominal wall on the arterial phase [29]. The average Hounsfield unit (HU) of the 3 ROIs was used as a representative value. Image noise was represented by the mean standard deviation (SD) of three measurements in the subcutaneous fat of the anterior abdominal wall. For contrast-to-noise ratio (CNR) and signal-to-noise ratio (SNR), the ROI was drawn on arterial phase images at the portal vein level, within a homogeneous area of the liver located between the middle and right hepatic veins. The ROI area ranged from 1 to 3 cm^2^ and was manually placed at three different locations. The ROI in the aorta was also measured. The CNR of focal arterial enhancing lesions were evaluated using the average value of the ROIs manually drawn at the 21 arterial enhancing lesions. The ROIs were drawn to include the lesion as much as possible. The SNR of the liver and CNR of the aorta were assessed on all 67 image pairs.

The SNR and CNR were calculated according to the following:

SNR of the liver = mean HU of the liver in the arterial phase/image noise.

CNR of the aorta = (mean HU of the aorta − mean HU of the subcutaneous fat)/image noise.

CNR of the focal arterial enhancing lesion = (mean HU of the focal arterial enhancing lesion − mean HU of the liver in the arterial phase)/image noise [25,28].

### 2.5. Focal Liver Lesion Evaluation

Two abdominal radiologists (*J.S.K.* with 13 years of experience in abdominal CT interpretation and *H.J.L* with 7 years of such experience) independently evaluated focal hepatic lesions showing enhancement in the arterial phase images. Their focus was on detectability, rating lesion conspicuity on a 5-point scale (Table 1). Lesions with a definite arterioportal (AP) shunt, characterized by wedge-shaped capsular enhancement, were excluded from the evaluation. To minimize recall bias, the reading sessions were separated by a washout period of 4 weeks. Every identified lesion was cross-referenced by one senior radiologist (*J.K.L.* with more than 20 years of experience) with clinical data including pathology results and findings from index and comparison images (CT, MR, or positron emission tomography/CT).

### 2.6. Statistical Analysis

Statistical significance was defined as two-tailed *p*-values < 0.05. Statistical analyses were conducted using commercially available software (IBM SPSS Statistics for Windows, v. 29.0; IBM, Armonk, NY, USA; or MedCalc, v. 19.2.1; MedCalc, Marikerke, Ghent, Belgium), except for the assessment of qualitative and focal hepatic lesion evaluation results. Continuous variables such as difference in patient characteristics and quantitative metrics including CNR and SNR were compared using a paired *t*-test and a paired Wilcoxon test for parametric data and non-parametric data, respectively.

The primary goal was the evaluation of the non-inferiority of the overall image quality of LCLM CT in arterial phases compared to SDCT. A pre-established non-inferiority margin of −0.5, as determined by prior studies [28,30], was used. Potential confounding factors in the overall image quality assessment were evaluated using a Generalized Estimating Equation (GEE) model.

To assess the agreement between the two inter-readers, Gwet’s agreement coefficient 2 (AC2) was calculated using the ‘irrCAC’ package (https://pypi.org/project/irrCAC/ accessed on 1 June 2025) in R [31]. A linear weighting scheme was applied to account for the ordinal nature of the data. For focal arterial enhancing lesion detectability evaluation, jackknife free-response receiver operating characteristic (JAFROC) analysis was employed to assess the figures of merit (FOM), including both individuals with and without arterial focal enhancing lesion [32]. The Dorfman–Berbaum–Metz multi-reader multicase method was applied using fixed-reader random-case output in JAFROC software (JAFROC, version 4.1; https://www.devchakraborty.com accessed on 1 January 2025.). Non-inferiority of LCLM CT compared to SDCT was established if the lower limit for the 95% confidence interval (CI) for the difference was greater than −0.1, a margin predefined based on similar previous studies [28,30,33].

## 3. Results

Overall, 67 patients (42 men and 25 women) with a mean age of 61.19 ± 12.00 years at examination (LCLM CT) were included in this study. The patients’ chronic liver disease etiologies were varied: twenty-four patients had chronic hepatitis B (35.8%), four patients had chronic hepatitis C (6.0%), twelve patients had alcoholic liver disease (17.9%), and twenty-seven patients had cryptogenic disease (40.3%). The time interval between LCLM CT and SDCT was 16.04 ± 22.01 months for all sixty-seven patients and 11.39 ± 7.36 months for the twenty patients with focal liver lesions. Three patients had >10% difference in body weight between the two CT scans; one patient experienced a 19.91% increase in body weight, while two patients lost 11.97% and 10.11% body weight, respectively. The laboratory findings, body weight, radiation dose, and iodine amount at each evaluation are described in Table 2. The amount of iodine per kilogram was significantly lower by 21.87% ± 4.43% in LCLM CT compared to SDCT (621.77 ± 101.96 vs. 485.16 ± 80.25, *p* < 0.001).

**Figure 1:**
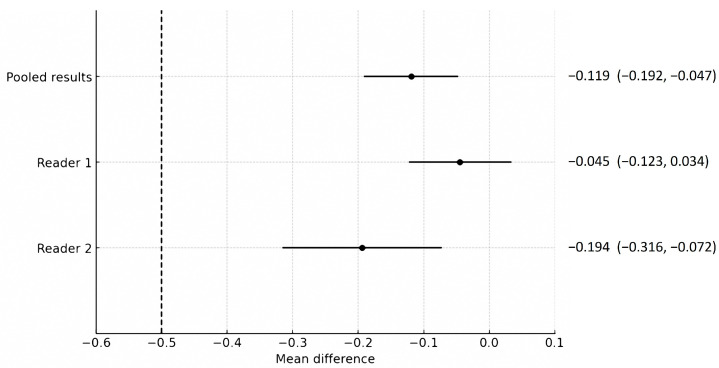
Comparison of the non-inferiority test results regarding the overall image quality of low-concentration iodine contrast using low-monoenergetic CT with that of standard-dose CT. The predefined non-inferiority margin of −0.5 is indicated by the dotted line. Each point and its horizontal line represent the mean difference and its 95% confidence interval (CI), with corresponding numerical values presented as the mean difference (95% CI) adjacent to the plot. Regarding the mean difference and 95% confidence interval for the pooled results, Reader 1 and Reader 2 did not cross the non-inferiority margin, demonstrating the non-inferiority of the low-concentration iodine contrast protocol.

Compared to SDCT, the overall image quality of LCLM CT, presented as the mean difference, was −0.119 (95% CI, −0.192–−0.047), which did not cross the predefined non-inferiority margin of −0.5 (*p* = 0.001) (Figure 1). Both groups had no suboptimal overall image scores (a score < 2). No significant association was found between overall image quality and scanner type (*p* = 0.066), sex (*p* = 0.349), age (*p* = 0.639), and body weight (*p* = 0.373), suggesting that these variables did not act as significant confounders. LCLM CT showed better scores than SDCT in hepatic clarity and liver contrast, while SDCT showed better scores than LCLM CT in image noise (Table 3 and Supplementary Table S2). Inter-reader agreement using Gwet’s AC2 ranged from 0.893 to 0.961, indicating high inter-reader agreement.

Noise was significantly higher in LCLM CT than in SDCT (12.45 ± 1.69 vs. 8.80 ± 2.77, *p* < 0.001). The measured average HU values of the liver, aorta, and focal liver lesion were significantly higher for LCLM CT than SDCT (*p* < 0.001, Table 4). The CNRs of the aorta and arterial enhancing focal lesions were also significantly higher for LCLM CT than SDCT (*p* < 0.001, Table 4 and Supplementary Table S3).

**Figure 2:**
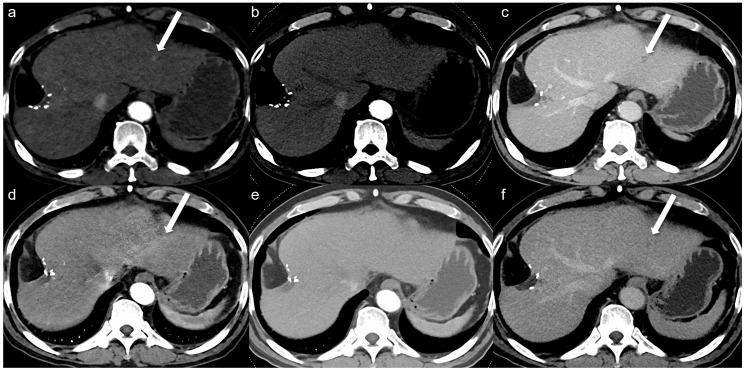
A 64-year-old man with hepatocellular carcinoma (HCC). Although the HCC was not detected by either reviewer on both the low-concentration iodine contrast-enhanced low-monoenergetic CT and the standard-dose CT, retrospective evaluation reveals the presence of an arterial enhancing lesion. Low-concentration iodine contrast using a low-monoenergetic CT and standard-dose CT were performed with a 3-month interval. In the low-concentration contrast medium (270 mg I/mL) monoenergetic 40 keV image (**a**), subtle arterial enhancement measuring approximately 9 mm is visible (arrow). However, arterial enhancement is not observed in the blended image (**b**) (a mixed ratio of 0.5, 50% 100 kV and 50% Sn140 kV). The portal phase image (**c**) reveals the washout (arrow). The standard-dose CT with 350 mg I/mL also showed subtle arterial enhancement in the monoenergetic image (**d, arrow**), which was not visible in the blended image (**e**) (a mixed ratio of 0.5, 50% 100 kV and 50% Sn140 kV) even though the portal phase washout was clearly seen (**f, arrow**). This lesion was confirmed as HCC on MRI with gadoxetic acid. This case illustrates that although low-monoenergetic CT images are associated with higher noise, the improved contrast-to-noise ratio can facilitate the detection of subtle arterial enhancing lesions.

**Figure 3:**
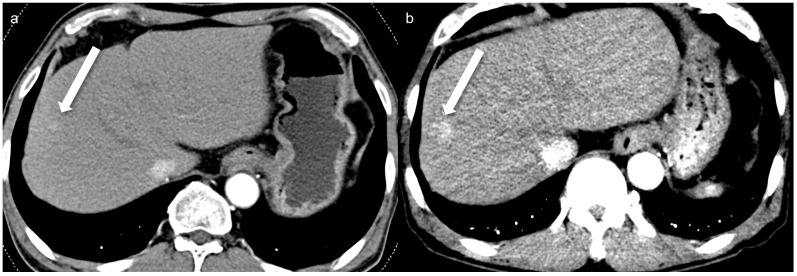
A 67-year-old man with an 8 mm nodular AP shunt. A prior standard-dose CT performed 4 months ago (**a**) shows subtle arterial enhancing nodular lesion (arrow), whereas a low-concentration contrast medium (270 mg I/mL) monoenergetic 40 keV image (**b**) shows more prominent arterial enhancement (arrow). The enhanced conspicuity on the monoenergetic image highlights the potential diagnostic benefit of monoenergetic 40 keV imaging in detecting subtle arterioportal shunts.

**Figure 4:**
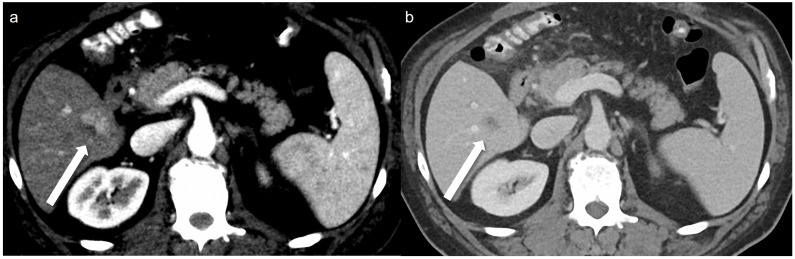
A 51-year-old man with 2 cm hepatocellular carcinoma (HCC). The low-concentration contrast medium (270 mg I/mL) monoenergetic 40 keV image (**a**) shows arterial enhancement (arrow), and the portal venous phase (**b**) shows washout (arrow).

A total of 32 arterial enhancing focal lesions were evaluated in 20 patients (27 lesions in SDCT and 28 lesions in LCLM CT). Of these, 23 lesions were identified as the same lesions in both SDCT and LCLM CT without significant interval change. These unchanged lesions were characterized as follows: 1 focal nodular hyperplasia, 3 HCCs, 5 hemangiomas, and 14 nodular AP shunts. Among the three HCCs, two were not detected in either CT scan but were confirmed on MRI with gadoxetic acid (Figure 2). The twenty-one unchanged arterial enhancing lesions (excluding the two non-detected HCCs) were evaluated for CNR (Figure 3). Four arterial enhancing lesions were only visible upon SDCT, and these were identified as HCC lesions treated with radiofrequency ablation and hepatectomy. Five arterial enhancing lesions were only visible upon LCLM CT, including three newly developed AP shunts, one HCC, and one cholangiocarcinoma (Figure 4). The mean lesion size was 12.84 ± 10.7 mm (SDCT, 12.78 ± 11.11 mm vs. LCLM CT, 13.04 ± 11.19 mm). The two HCCs that were not detected on both SDCT and LCLM CT were not detected by either reader. During SDCT, reader 1 could not detect one nodular AP shunt, whereas reader 2 could not detect two nodular AP shunts. All of the other lesions were detected by both readers. The mean size of undetected lesions was 8.60 ± 8.23 mm, while detected lesions measured 15.07 ± 10.76 mm. However, this difference was not statistically significant (*p* = 0.213). Lesion conspicuity was not significantly different between SDCT and LCLM CT (SDCT, 4.37 [95% CI: 3.94–4.80] vs. LCLM CT, 4.51 [95% CI 4.11–4.91]) (*p* = 0.293). The FOM for detectability of the arterial hepatic focal lesion was slightly higher in LCLM CT (FOM, 0.744) than that of SDCT (FOM, 0.721) without statistical difference (difference: 0.023, 95% CI: −0.170–0.218) (Supplementary Tables S4 and S5). As the 95% CI crosses the predefined margin of −0.1, non-inferiority is not statistically confirmed.

## 4. Discussion

LCLM CT, with a 21.87% reduction in iodine concentration using low-iodine contrast, demonstrated its non-inferiority for overall image quality (difference: −0.119, 95% CI: −0.192–−0.047) compared to SDCT. In quantitative analysis, the CNRs of the aorta and arterial enhancing focal lesions were significantly higher in LCLM CT than in SDCT.

Our findings are consistent with previous studies demonstrating the ability of monoenergetic images to reduce iodine concentration by increasing the CNR [17,25,34]. However, most prior studies focused on reducing the dose of contrast agents [25,35]. Conversely, our approach aimed to reduce the total amount of iodine by lowering its concentration. Lowering the iodine concentration can not only decrease viscosity and osmolality but also reduce the peak injection pressure associated with extravasation risks [11,14]. A previous study showed that using low-concentration contrast in low-tube voltage CT urography was non-inferior to high-concentration contrast CT [36]. However, research on reducing iodine concentration to decrease the iodine amount in hepatic multiphase CT is limited. Other studies reduced iodine concentration by using deep learning reconstruction to adjust the iodine amount [37]. A recent study on patients with reduced estimated glomerular filtration rates has shown that the results were non-inferior to those with the standard-dose CT when using a lower iodine concentration (320 mg I/mL), a low iodine dose (300 mg I/kg), monoenergetic imaging (50 keV), and a deep learning–based iodine boosting method [38]. However, our goal was to determine whether lowering the iodine concentration with monoenergetic imaging using dual-energy CT could maintain diagnostic performance and image quality given that deep learning reconstruction is not always available. In our study, the CNR of LCLM CT was significantly higher than that of SDCT (CNR of the aorta, SDCT vs. LCLM CT, 51.69 ± 18.43 vs. 83.36 ± 18.86, *p* < 0.001; CNR of arterial enhancing focal lesions, 9.27 ± 5.74 vs. 17.99 ± 10.68, *p* < 0.001). Furthermore, our study focused on patients with chronic liver disease undergoing hepatic multiphase CT who are at an increased risk of contrast-related adverse effects due to frequent imaging. Therefore, performing CT that uses monoenergetic imaging with reduced iodine concentration and burden could be helpful in preserving renal function throughout the long follow-up period. Since LCLM CT does not incur a significant increase in cost or image acquisition and processing time compared to SDCT, this clinical advantage is thought to be potentially more meaningful.

Our study showed that the detectability (LCLM CT vs. SDCT, 0.744 vs. 0.721) and conspicuity (SDCT, 4.37 [95% CI: 3.94–4.80] vs. LCLM CT, 4.51 [95% CI 4.11–4.91], *p* = 0.293) of LCLM CT for focal hepatic artery enhancing lesions were not lower than those of SDCT. Previous studies on rabbits also showed that using monochromatic imaging could improve the image quality by reducing noise and increase the detection rate of small tumors when comparing 270 mg I/mL to 370 mg I/mL [21]. Most studies on patients using low-concentration contrast media in multiphase CT used the iodine concentration of ≥300 mg/mL [13,38]. Because lower iodine concentration contrast may affect the accuracy of bolus tracking, a previous study suggested that arterial enhancement, particularly in patients with chronic liver disease, may be reduced [39]. Given that HCC requires the detection of subtle arterial enhancement, a more refined approach may be necessary for patients with chronic liver disease. Therefore, we focused on the evaluation of the arterial phase. The detectability of arterial-enhancing lesions was assessed using a figure-of-merit approach, and the non-inferiority was not statistically confirmed (difference in FOM: 0.023, 95% CI: −0.170–0.218), although the FOM of LCLM CT was slightly higher than that of SDCT. This analysis was exploratory in nature, and the study was not statistically powered to test non-inferiority for this endpoint.

Despite the significant improvement in CNR, the higher noise in arterial phase images using monoenergetic imaging was a notable limitation (12.45 ± 1.69 vs. 8.80 ± 2.77, *p* < 0.001). While the significant improvement in CNR may potentially aid in identifying small or subtle hypervascular lesions by making the lesions more discernible relative to the background noise, the elevated noise levels carry potential risks. They could compromise the detection of subtle lesions such as early HCCs and may be more problematic in patients with heterogeneous liver backgrounds. These factors may increase the risk of false negatives in specific subgroups such as obese patients with diffuse fatty liver, thus warranting careful interpretation in this clinical context. Although noise increases at lower keV levels, our results demonstrate that arterial enhancement remains comparable, thereby supporting the feasibility of hepatic multiphase CT with low-concentration iodine contrast agents in clinical practice using monoenergetic imaging. This approach offers the potential to reduce total iodine exposure without significantly compromising diagnostic performance. For clinical deployment of LCLM CT, further large-scale studies across various lesion types and sizes are warranted, along with consideration of factors such as cost and limited availability of dual-energy CT scanners and software licensing. Nevertheless, our study can serve as an important starting point that may lay the groundwork for future research in this area.

Previous studies have demonstrated that deep learning reconstruction techniques could reduce noise in monoenergetic imaging, which may address the current limitation of higher noise levels [13,37]. Moreover, a recent study demonstrated that using deep learning-based denoising techniques on LCLM CT images showed comparable HCC conspicuity and detection [35]. Therefore, applying deep learning reconstruction to monoenergetic images may be a potential solution to current limitations and enhance its clinical applicability. However, several challenges such as availability, technical feasibility, algorithm validation, and generalizability, along with regulatory considerations, remain as key tasks to be addressed.

This study has several limitations. First, it was a single-center retrospective study sequentially comparing LCLM CT with an earlier SDCT in the same patient. Given the time interval between the two scans, the lesion itself might change, potentially influencing the differences between LCLM CT and SDCT images and introducing bias. While previous studies compared monoenergetic imaging to conventional imaging performed in different patients, our study benefits from comparing images of the same lesion in the same patient, potentially reducing inter-patient variability. Also, no significant change in lesion size was observed between the two scans (SDCT, 12.78 ± 11.11 mm vs. LCLM CT, 13.04 ± 11.19 mm, *p* = 0.086), suggesting that the risk of temporal bias may have been minimal. However, it is important to acknowledge that the results may be susceptible to potential interference due to differences in timing. The patients in our study were randomly assigned to undergo CT scans on one of two CT scanners at our institution, with only one of them being a dual-energy CT scanner and the other a single-energy CT scanner. Due to the random assignment, we believe the possibility of selection bias is small, and the differing CT scanners did not act as a statistically significant confounder in our cohort (*p* = 0.066). Nonetheless, further large-scale prospective studies designed to be free of both temporal and selection bias would help in evaluating and generalizing the application of LCLM CT. Second, this study was performed with only one vendor (Siemens Healthineers), possibly affecting the generalizability of the results. Therefore, further testing with different vendors and CT protocols is needed to assess the broader applicability. However, all scans were performed using standardized acquisition protocols within each system, and image analysis was performed in an intra-individual design to minimize inter-patient variability. Third, the wide confidence intervals of FOM for lesion detectability suggest that the reliability of the results should be interpreted with caution. Although lesion detectability was assessed using an FOM approach, this analysis was exploratory in nature, and the study was not statistically powered to test this endpoint. The sample size was calculated prior to the study, and it was determined that at least 41 patients would be sufficient. However, the relatively small sample size limits the generalizability of the findings, particularly to various subgroups such as lesion type, size, or patient risk profile. Therefore, further larger-scale studies are needed to enable comprehensive comparisons of detectability across different lesion characteristics and imaging modalities and confirm the findings of the present study. Fourth, the intrinsic difference in image reconstruction techniques between groups serves as a notable limitation: monoenergetic reconstruction was used in the LCLM CT group, while blended dual-energy images were utilized in the SDCT group. Differences can arise due to variations in acquisition techniques, material decomposition methods, image reconstruction algorithms, and postprocessing procedures [40]. These differences reflect fundamentally distinct image properties that cannot be fully addressed through statistical adjustment. Therefore, comparisons between the two groups should be interpreted with caution. In conclusion, hepatic multiphase LCLM CT using 270 mg I/mL iodine contrast and 40 keV monoenergetic imaging demonstrated comparable image quality and lesion detectability to standard 350 mg I/mL SDCT in our limited cohort. This protocol has the potential to reduce iodine load by approximately 20% without markedly compromising arterial phase lesion detection, which may be beneficial for chronic liver disease patients requiring repeated imaging. However, the observed limitation in lesion detectability due to increased image noise observed at low keV levels, combined with the single-center, retrospective nature of this study, highlights the need for further validation in larger and more diverse patient populations before broader clinical adoption, particularly in the context of long-term surveillance of hypervascular liver lesions.

## Figures and Tables

**Table 1:** Qualitative image evaluation criteria.

Score	Overall Image Quality	Hepatic Artery Clarity	Contrast of the Liver	Image Noise	Lesion Conspicuity
1	Very poor (non-diagnostic quality and reexamination needed)	Not delineated	Substantial lack of contrast similar to non-contrast CT or the nephrogenic phase	Non-diagnostic images with severe noise	Not detectable
2	Suboptimal (unsatisfactory quality but reexamination not needed)	Barely distinct	Poor contrast	Moderate noise with diagnostic performance impairment	Barely distinct
3	Average	Clear common hepatic artery (CHA) and proper hepatic artery (PHA) but blurred HA	Average contrast	Moderate noise but no diagnostic performance impairment	Moderately distinct (blurry margin)
4	Above average	Bilateral HA is clearly visible, but segmental HA is blurred	Good contrast	Mild noise without effect on the diagnostic quality	Fairly distinct (round but some blurry margin)
5	Excellent	The entire HA (including segmental branch) is clearly visible	Very strong contrast	No perceivable noise	Definitely distinct (with sharp margin)

**Table 2:** Demographics of the study population (*n* = 67).

Characteristics	SDCT	LCLM CT	*p*-Value
**Laboratory findings**			
Albumin, g/dL	4.19 ± 0.65 (2.5–5.0)	4.29 ± 0.58 (1.9–5.1)	0.186
Total bilirubin, mg/dL	1.35 ± 2.27 (0.33–14.69)	1.02 ± 0.88 (0.23–4.90)	0.200
Platelet count, ×103/mm^3^	195.49 ± 109.65 (47–650)	188.81 ± 85.33 (40–416)	0.480
AFP, ng/mL	32.83 ± 121.25 (1–663)	21.32 ± 91.35 (1–634)	0.295
**Body weight, kg**	68.06 ± 12.03 (35–100)	67.27 ± 12.22 (35–100)	0.124
**Mean body mass index, kg/m^2^**	25.01 ± 3.73 (13.50–35.61)	24.61± 3.49 (13.50–32.92)	0.020
**Iodine dose per kilogram**	621.77 ± 101.96	485.16 ± 80.25	<0.001
**DLP, mGycm**	1001.59 ± 367.22 (318–2216)	880.03 ± 329.90 (329–1837)	<0.001
**Effective dose, mSv**	15.02 ± 5.51 (4.77–33.24)	13.30 ± 4.94 (4.94–27.56)	<0.001

Note: Data are presented as mean ± standard deviation, with the range (minimum–maximum) in parentheses. The unit of each parameter is noted after the comma of each criterion. Abbreviations: AFP, alpha-fetoprotein; DLP, dose length product.

**Table 3:** Qualitative image quality analysis between standard-dose CT and low-concentration iodine contrast low-monoenergetic CT of arterial phase images.

	SDCT	LCLM CT	*p*-Value	Inter-Reader Agreement (Gwet’s AC2)
Overall image quality	4.94 ± 0.24 [4.10, 5.78]	4.82 ± 0.46 [4.00, 5.64]	0.001	0.929 [0.893, 0.965]
Hepatic artery clarity	4.83 ± 0.50 [4.01, 5.64]	4.93 ± 0.32 [4.09, 5.76]	0.047	0.961 [0.932, 0.985]
Contrast of the liver	4.90 ± 0.31 [4.07, 5.72]	4.97 ± 0.24 [4.13, 5.81]	0.032	0.959 [0.932, 0.985]
Image noise	4.93 ± 0.25 [4.10, 5.77]	4.83 ± 0.42 [4.01, 5.65]	0.002	0.893 [0.848, 0.937]

Note: Data are presented as mean ± standard deviation, with 95% confidence intervals in brackets. Abbreviation: SDCT, standard-dose CT; LCLM CT, low-concentration iodine contrast low-monoenergetic CT.

**Table 4:** Quantitative analysis between standard-dose CT and low-concentration iodine contrast low-monoenergetic CT of arterial phase images.

Quantitative Analysis	SDCT	LCLM CT	*p*-Value
Noise	8.80 ± 2.77	12.45 ± 1.69	<0.001
Measured average HU			
Liver	70.27 ± 11.91	97.38 ± 17.62	<0.001
Aorta	324.19 ± 106.46	835.06 ± 179.22	<0.001
Focal liver lesion	147.63 ± 51.78	330.90 ± 152.46	<0.001
SNR of the liver	8.63 ± 2.55	7.97 ± 1.78	0.058
CNR of the aorta	51.69 ± 18.43	83.36 ± 18.86	<0.001
CNR of arterial enhancing focal lesions	9.27 ± 5.74 ^†^	17.99 ± 10.68 ^†^	<0.001

Abbreviation: SDCT, standard-dose CT; LCLM CT, low-concentration iodine contrast low-monoenergetic CT; HU, Hounsfield unit; SNR, signal-to-noise ratio; CNR, contrast-to-noise ratio. † values for CNR were measured in 21 arterial enhancing lesions.

## Data Availability

The data presented in this study are available upon request from the corresponding authors.

## Supplementary Materials

The following supporting information can be downloaded at: https://www.mdpi.com/article/10.3390/tomography11060066/s1, Table S1. Image acquisition of different CT scanners; Table S2. Image quality values of SDCT and LCLM CT with different scanners; Table S3: Quantitative analysis of SDCT with or without iterative reconstruction; Table S4. Figure-of-merit values of SDCT with or without iterative construction; Table S5. Figure-of-merit values of SDCT with different scanners.

## Abbreviations

The following abbreviations are used in this manuscript:

CT	Computed tomography
SDCT	Standard-dose computed tomography
LCLM CT	Low-concentration iodine contrast-enhanced multiphase low-monoenergetic computed tomography
DECT	Dual-energy computed tomography
SNR	Signal-to-noise ratio
CNR	Contrast-to-noise ratio
CI	Confidence interval
HCC	Hepatocellular carcinoma
ROI	Region of interest
FOM	Figures of merit

## Appendix A. Sample Size Confirmation

To assess the non-inferiority of overall image quality of LCLM CT, sample size ample size calculation was based on a margin of non-inferiority for image quality score set at −0.5 of 5-point scale analysis. This margin was selected based on that used in a previous non-inferiority reference study [25,28] and elsewhere in the literature [41]. Standard deviation of mean difference (0.5) was adopted from a previous study [25], with a power of 80% and a one-sided α-level of 0.05, we estimated that 41 patients were needed to show the non-inferiority of the low-concentration iodine contrast monoenergetic imaging.
