# Supplementary material for: Image Quality and Lesion Detectability with Low-Monoenergetic Imaging: A Study of Low-Concentration Iodine Contrast in Hepatic Multiphase CT for Chronic Liver Disease
Source: Tomography. 2025 Jun 4;11(6):66. doi: 10.3390/tomography11060066 (PMC12197165; doi:10.3390/tomography11060066)
Supplement: Supplementary file 1 [file tomography-11-00066-s001.zip › tomography-3547186-supplementary.pdf]

Table S1. Image acquisition of different CT scanners.

Parameter	SOMATOM Force	SOMATOM Definition Flash	SOMATOM Sensation 64
**Number of Detector Rows**	192	128	64
**Collimation**	128 × 0.6 mm	32 × 0.6 mm	32 × 0.6 mm
**Pitch**	0.6	0.6	1
**Reconstruction Interval**	3 mm	3 mm	3 mm
**Image Acquisition Type**	Dual-Energy (Mixed Image)		Single-Energy
**Tube Voltage**	80 kV and tin filtered 150 kV (Sn150 kV)	100 kV and tin filtered 140 kV (Sn140 kV)	100 kV
**Reference Tube Current**	325/163 mAs (80 kV/Sn150 kV)	219/170 mAs (100 kV/Sn140 kV)	210 mAs

Table S2. Image quality values of SDCT and LCLM CT with different scanners

Qualitative analysis	SDCT	LCLM CT	Difference
Sensation 64 (n = 20)	4.85 ± 0.362	4.60 ± 0.672	-0.250 (95% CI –-0.438 - 0.620)
Somatom Flash (n = 38)	4.97 ± 0.161	4.89 ± 0.309	-0.079 (95% CI –-0.160 - 0.002)
Somatom Force (n = 9)	5.00 ± 0.00	5.00 ± 0.00	N/A

Abbreviation: SDCT, standard-dose CT; LCLM CT, low-concentration iodine contrast low monoenergetic CT; N/A, non-applicable.

Note: Difference means difference of image quality was obtained by image quality using low concentration iodine contrast low monoenergetic CT images subtracted by image quality using SDCT.

Table S3. Quantitative analysis of SDCT with or without iterative reconstruction

Quantitative analysis	SDCT with iterative reconstruction (n = 49)	SDCT without iterative reconstruction (n = 18)	p-value
Noise	7.37 ± 1.21	12.70 ± 1.88	<0.001/ 0.934
SNR of the liver	9.67 ± 2.05	5.80 ± 1.34	<0.001/ <0.001
CNR of the aorta	57.34 ± 17.97	36.30 ± 7.92	< 0.001/ <0.001
CNR of arterial enhancing focal lesions	10.13 ± 10.94[†]	4.11 ± 2.11[‡]	0.001/ 0.162

Abbreviation: SDCT, standard-dose CT; SNR, signal-to-noise ratio; CNR, contrast-to-noise ratio.

Note: The first p-value is from a comparison between SDCT with iterative reconstruction and low-concentration iodine contrast low monoenergetic CT. The second p-value is from a comparison between SDCT without iterative reconstruction and low-concentration iodine contrast low monoenergetic CT.

[†] Values for CNR were measured in 18 arterial enhancing lesions.

[‡] Values for CNR were measured in 3 arterial enhancing lesions.

Table S4. Figure-of-merit values of SDCT with or without iterative reconstruction

	SDCT with iterative reconstruction	SDCT without iterative reconstruction
Figure of merit	0.720 (95% CI 0.606–0.834)	0.718 (95% CI 0.469–0.968)
Difference	0.017 (95% CI −0.204–0.238)	0.056 (95% CI −0.442–0.555)

Abbreviation: SDCT, standard-dose CT.

Note: Difference means difference of figure-of-merit of detectability was obtained by figure-of-merit using low concentration iodine contrast low monoenergetic CT images subtracted by figure-of-merit using SDCT.

Table S5. Figure-of-merit values of SDCT with different scanners

	SDCT with SOMATOM Definition Flash	SDCT with SOMATOM Force
Figure of merit	0.699 (95% CI 0.590–0.809)	0.738 (95% CI 0.518–0.957)
Difference	0.036 (95% CI −0.176–0.249)	0.000 (95% CI −0.437–0.437)

Abbreviation: SDCT, standard-dose CT.

Note: Difference means difference of figure-of-merit of detectability was obtained by figure-of-merit using low concentration iodine contrast low monoenergetic CT images subtracted by figure-of-merit using SDCT.
